# Characterization of two *ETFDH* mutations in a novel case of riboflavin-responsive multiple acyl-CoA dehydrogenase deficiency

**DOI:** 10.1186/s12944-018-0903-5

**Published:** 2018-11-13

**Authors:** Sara Missaglia, Daniela Tavian, Laura Moro, Corrado Angelini

**Affiliations:** 1 Laboratory of Cellular Biochemistry and Molecular Biology, CRIBENS, Catholic University of the Sacred Heart, pz Buonarroti 30, 20145 Milan, Italy; 2 Psychology Department, Catholic University of the Sacred Heart, Largo Gemelli 1, 20123 Milan, Italy; 3 Department of Pharmaceutical Sciences, University of Piemonte Orientale, Lgo Donegani 2, 28100 Novara, Italy; 4 Fondazione Ospedale San Camillo IRCCS, via Alberoni 70, 30126 Venice, Italy

**Keywords:** Multiple acyl-CoA dehydrogenase disorder, Lipid storage myopathy, ETFDH, Carnitine, Riboflavin

## Abstract

**Background:**

Deficiency of electron transfer flavoprotein dehydrogenase (ETFDH) is associated with multiple acyl-CoA dehydrogenase deficiency (MADD). This disorder is an autosomal recessive lipid storage myopathy (LSM) that exhibits a wide range of clinical features, including myopathy, weakness and multisystem dysfunctions. Many patients with late onset of MADD improve when treated with riboflavin and are also referred to as RR-MADD (riboflavin-responsive multiple Acyl-CoA dehydrogenase disorder).

**Methods:**

In this study, we report the clinical and genetic characterization of a novel RR-MADD patient. Biochemical data were obtained from analysis of muscle and plasma samples. DNA and RNA were extracted from peripheral blood, and sequence analysis and expression study of *ETFDH* gene were performed. Finally, the impact of mutations on ETFDH folding was evaluated using bioinformatic tools.

**Results:**

Patient initially presented with vomiting, muscle weakness, and acidosis. Muscle biopsy revealed typical myopathological patterns of lipid storage myopathy and blood acylcarnitine profiles showed a combined elevation of long and medium chain acylcarnitines, supporting the diagnosis of RR-MADD. Molecular analysis of *ETFDH* gene revealed two heterozygous mutations, a novel splice variation in intron 10, c.1285 + 1G > A, and the previously reported c.560C > T missense mutation. RT-PCR analysis showed an alteration of *ETFDH* RNA splicing which in turn should lead to the production of a truncated protein. The in silico prediction analysis of ETFDH tridimensional structure demonstrated that the missense mutation resulted in instability and loss of protein activation, while the splice site variation induced a dramatic conformational change of the truncated protein. After MCT diet supplemented with carnitine and riboflavin, the patient showed significant biochemical and clinical improvement, in spite of severe molecular defect.

**Conclusion:**

This case report extends the spectrum of ETFDH mutations in MADD, providing further evidence that patients presenting at least one missense mutation in the FAD-binding domain may respond to either carnitine or riboflavin treatment, due to the recovery of some enzymatic activity.

## Background

**Fig. 1:**
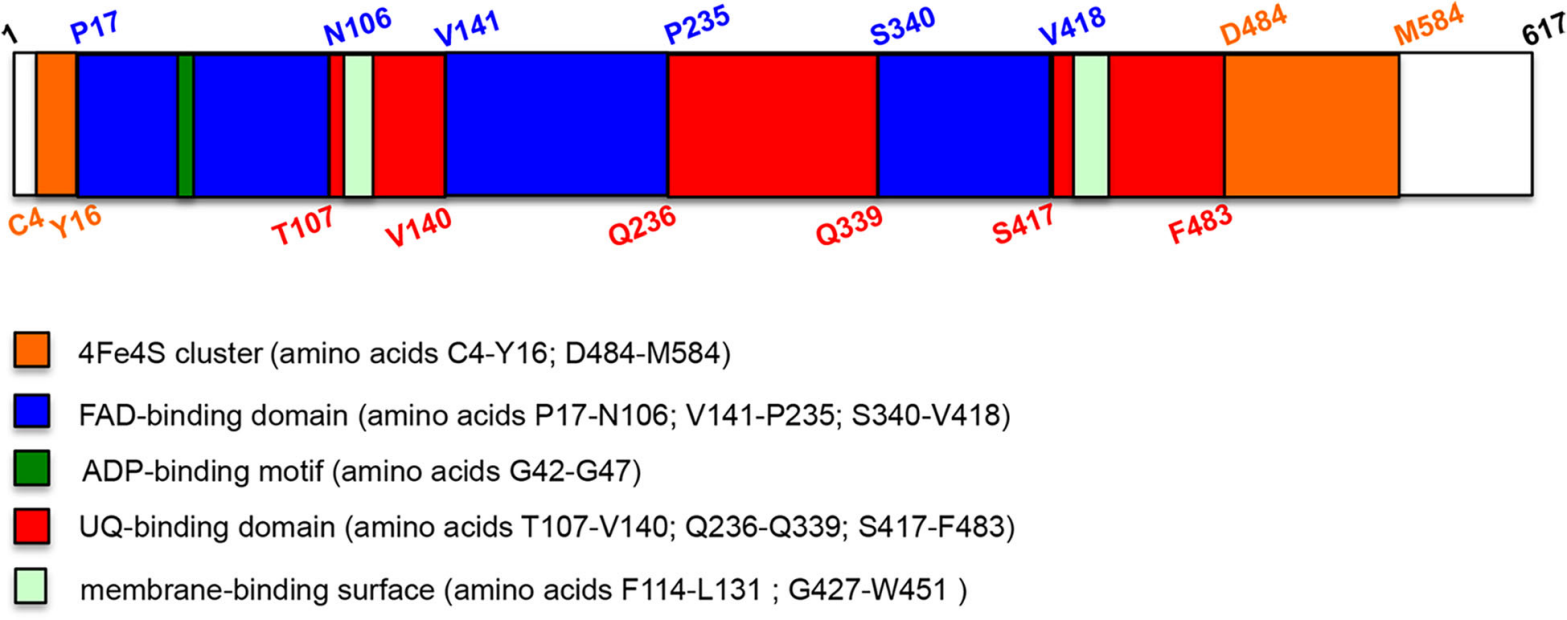
Schematic representation of ETFDH structural domains. ETFDH protein (amino acids 1–617) has three functional regions: the 4Fe4S cluster (amino acids C4-Y16 and D484-M584; the FAD-binding domain (amino acids P17-N106, V141-P235 and S340-V418), that contains in the first segment, between G42-G47 residues, an ADP-binding motif; the UQ-binding domain (amino acids T107-V140, Q236-Q339 and S417-F483). Furthermore, two membrane-binding surface regions are located in the UQ domain

Electron transfer flavoprotein dehydrogenase (ETFDH), also called ETF-ubiquinone oxidoreductase, is a mitochondrial protein localized in the inner membrane, that plays a key role in the electron-transfer system [1]. In particular, ETFDH mediates electron transport from flavoprotein dehydrogenases to the ubiquinone pool [2]. This protein is codified by *ETFDH* gene localised on chromosome 4, and consists of 617 aa residues. Data of ETFDH structure, obtained from x-ray crystallography analysis [1], showed that this protein possesses three, nearly connected, functional regions: FAD-binding domain, 4Fe4S cluster and ubiquinone (UQ) binding domain (Fig. 1). Moreover, an ADP-binding motif is localized in the FAD-binding domain (between amino acids G42-G47) and two membrane-binding surface regions are identified within the UQ binding domain (aa residues F114-L131 and G427-W451).

Mutations of *ETFDH* cause the onset of Multiple acyl-CoA dehydrogenase deficiency (MADD). MADD, also known as Glutaric aciduria type II (MIM 231680), is a rare autosomal recessive inherited disorder of fatty acid, amino acid, and choline metabolism. MADD is associated with mutations not only in *ETFDH,* but also in *ETFα*, *ETFβ*, *SLC52A1* and *FLAD1* [3–8]. The genetic heterogeneity correlates with different clinical phenotypes that can be divided into three types [9]: 1) neonatal onset with congenital anomalies (MADD type I). The clinical features of this group are non-ketotic hypoglycemia, hypotonia, hepatomegaly, metabolic acidosis, dysplastic kidneys, facial dysmorphism, rocker-bottom feet and anomalies of external genitalia. The symptoms appear during the first 24 h of life and patients usually die within the first week of life; 2) neonatal onset without anomalies (MADD type II). Hypotonia, tachypnea, hepatomegaly, metabolic acidosis and hypoketotic hypoglycemia arise within the first 24–48 h of life and the death often occurs within the first weeks of life; 3) mild and/or late onset (MADD type III). In this case, the patients show a variable age of disease onset and different clinical symptoms: lipid storage myopathy, cardiac damage, intermittent episodes of vomiting and metabolic acidosis. The majority of MADD subjects with mild and/or late-onset harbor mutations in the *ETFDH* gene. Most of the patients presenting MADD type III are responsive to riboflavin supplementation and, for this reason, the clinical phenotype is also called riboflavin-responsive MADD (RR-MADD) [10]. Moreover, the treatment can include a low-fat diet with carnitine and MCT supplementation [8, 9, 11].

In this study, we report the molecular and clinical findings of a 54-year-old woman affected by RR-MADD that presented fluctuating weakness since childhood. Genetic investigation showed that patient was compound heterozygotes for the c.560C > T and c.1285 + 1G > A mutations. Expression analysis demonstrated that splice site mutation produces an aberrant RNA. Finally, 3D structure comparison between ETFDH wild-type and mutant proteins displayed substantial changes in the protein folding, especially in the FAD-binding domain.

## Methods

### Biological samples

DNA and RNA samples were extracted from peripheral blood of the patient and control subject after obtaining informed consent for genetic analysis. Furthermore, written informed consent was obtained from the patient for publication of the article and of any accompanying image.

### Biochemical analysis

Carnitine and its fractions (acyl-carnitines) were extracted from muscle and plasma. Muscle biopsy was obtained from patient and it was frozen immediately, according to methods previously described by Chapoy et al. [12]. The levels of free carnitine were measured using a standardized radiochemical method in the presence of 0.5 mM N-ethylmaleimide [13]. Short and long-chain acylcarnitine were determined during alkaline hydrolysis [14]. Organic acids profile was investigated by mass spectrometry. Mitochondrial respiratory chain enzymes activity (OX-PHOS) was measured by standard spectro photometric methods described in Angelini et al. [15].

### Muscle biopsy

Muscle biopsies were used for histopathological evaluation, following a panel of routine histochemical and histoenzymatic stains.

### *ETFDH* exons analysis

Genomic DNA was extracted from peripheral blood of the patient and of a control subject using Puregene DNA Isolation kit (Qiagen). To amplify *ETFDH* exons sequences and their flanking regions, primers and PCR conditions were used as previously reported [16]. All PCR products were purified using NucleoSpin Extract kit (MACHEREY-NAGEL) and sequenced on 3730 DNA Analyzers by the BigDye® Terminator V1.1 Cycle Sequencing Kit (Applied Biosystems).

### RT-PCR analysis of *ETFDH* expression in peripheral blood

RNA was isolated from a frozen blood samples with TRIzol (Life Technologies), following the extraction procedure as described in Kim et al. [17]. 1 μg of RNA, treated with DNase I (ThermoScientific), was converted to cDNA by RT-PCR [18]. 50 ng of cDNA were used to perform PCR amplification using ETFDH-10cF (5’-CCTGGTGGTTTACTAATTGG-3′) and ETFDH-10R (5’-AATCTTTTAATGTCAATTGAA -3′) primers to detect intron retention and ETFDH-10cF (5’-CCTGGTGGTTTACTAATTGG-3′) and ETFDH-11cR (5′- TTTCTAACAGAATATAGCTC -3′) to produce a control fragment. PCR conditions for ETFDH-10cF/10R and ETFDH-10cF/11cR primers were as follows: first cycle consisting in denaturation at 95 °C for 3 min, annealing at 50 °C for 30 s and extension at 72 °C for 30 s; 28 cycles comprising denaturation at 94 °C for 30 s, annealing at 50 °C for 30 s and extension at 72 °C for 30 s; last cycle consisting in denaturation at 94 °C for 30 s, annealing at 50 °C for 30 s and final extension at 72 °C for 3 min.

### In silico analysis of ETFDH mutations

ClustalW, SIFT and PolyPhen software were used to predict the effect of p.A187V mutation on protein function. The NCBI reference sequence (NP_001268666.1) of the human ETFDH protein was used as the input.

Two bioinformatic tools, I-Tasser and SWISS-MODEL, were used to generate ETFDH wild-type, ETFDH(A187V) and ETFDH(G429Dfs21*) 3D models. The porcine ETFDH structure, obtained from x-ray crystallography analysis, was considered as a model template as it shares 95% of homology with human aa sequence.

## Results

### Case presentation

**Fig. 2:**
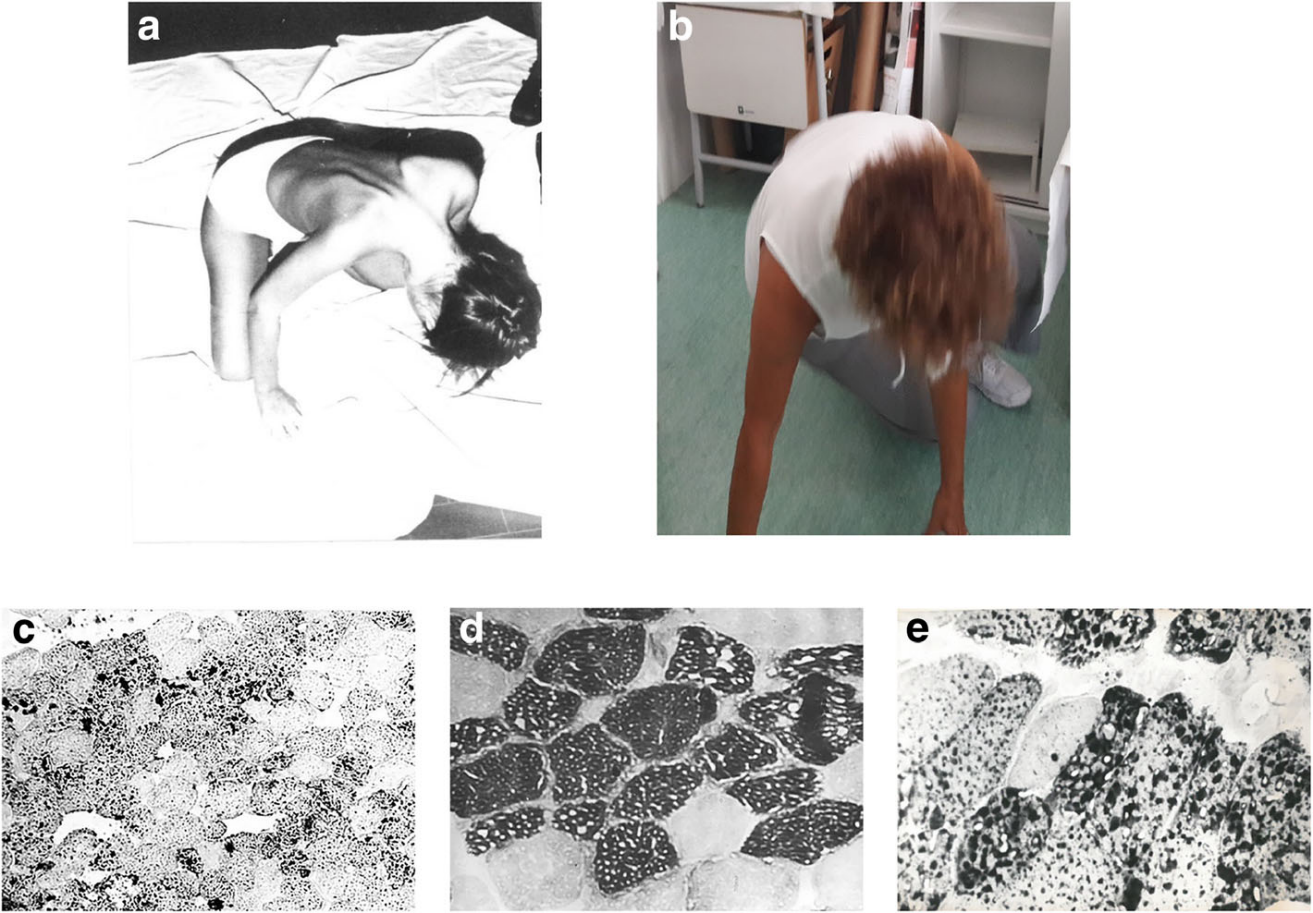
Clinical phenotype of RR-MADD patient. **a** Patient at age 12 shows marked neck and trapezius muscle weakness with dropping head; **b** Patient at age 54 raising from floor has better head control in Gowers manoeuvre; **c** Sudan Black B stain shows positivity in muscle biopsy at age 12; **d** with acid ATPase type I fibers appear vacuolated and (**e**) with Sudan black B stain vacuoles appear positive in a muscle biopsy performed at age 15

The proband was a 54-year-old patient with a story of muscle weakness and exercise intolerance, suggestive of lipid storage myopathy. Her symptoms started at two years of age when recurrent episodes of vomiting, drowsiness, appetite loss, asthenia and acetonemic breath appeared. At six years of age, she complained of the progressive arm, lower limb and neck flexors weakness, so that she was unable to rise from the floor, climb stairs and abduct her arms. An EMG showed signs of protopathic myopathic pattern and elevated plasma CPK (950 U/L) and LDH (877 U/L). The patient was first given Glycine 3 g/die, Vitamins and Mestinon 50 mg/die. After a beach vacation, the girl recovered her muscular strength for a period of about three months. Back to school, she presented an aggravation of muscular weakness particularly in hand muscle (difficulty in writing) and of the face (difficulty in chewing and articulating words). In the following period, for 6 months, she followed physiotherapy, which allowed a remarkable functional recovery. At 10 years, however, the muscle symptomatology reappeared, with a rapid return to previous conditions. On this occasion, vomit, appetite loss, acetonemic breath also reappeared. For these reasons, she was admitted to the University Hospital of Padua, where a muscle biopsy was performed, showing an intracellular accumulation of triglycerides (Fig 2c). EMG of the triceps and biceps brachii revealed neurogenic signs, likely representing the involvement of terminal nerve branches. Moreover, ECG evidenced non-specific modifications of repolarization. Serum and urinary determinations of carnitine were below normal (3.74 micromol/dL and 11.408 micromol/24 h, respectively). She was discharged with a diagnose of carnitine deficiency myopathy and supplemented with DL-carnitine therapy for 6 g/day and MCT diet and physical therapy recommended. This therapy led to a noticeable and progressive improvement in muscle strength and trophism; after 10 days the patient managed to stand up, after 20 days she climbed the stairs and went to school. One year later, she suspended the therapy because DL-carnitine determined the appearance of 4–5 liquid discharges per day. Only after one month, she had difficulty to move from the supine to the sitting position, weakness of the trunk muscles and external rotators of the arms (Fig 2a). Therapy was again prescribed with DL-carnitine per os in doses of 4 g/day and MCT diet, following which the patient significantly improved, so she was able to make long excursions on foot or ride a bike. At the age of 15, the patient performed a control of serum enzymes, which were high: CPK 465 mU / mL (n.v. up to 60), Aldolase 8.1 mU / mL (n.v. 0.5–3.1), LDH 378 mU / mL (n.v. up to 275). In the following years, the patient performed several hospitalizations, following the appearance of the usual diffuse asthenia, particularly in the lower limbs and muscular pain. Among the diagnostic investigations, we carried out also a liver biopsy, showing mild swelling of hepatocytes and a second muscle biopsy (Fig 2d and e), revealing less LSM.

On supplemental oral carnitine, the patient had significant acylcarnitine elevations as demonstrated by muscle, plasma and urinary carnitine dosages (Table 1). Since the age of 36, after the last admission, the patient reports feeling better and being able to carry out daily activities without problems (Fig. 2b). Since then, she follows a hypolipidic diet supplemented with MCT oil and a therapy based on Carnitine 2 g and Riboflavin 100 mg cp/day.

**Table 1:** Laboratory results of our patient

Age	Test	Results	Normal Values	Units
24 years	Urine TC	99.94	83.1–777.8	nMol/mg protein
	Muscle TC	4.23*	10.5–29.5	nMol/mg protein
26 years	Urine TC	1335.0*	36–561.7	mMol/24 h
	Urine FC	454.5*	30.5–282.1	mMol/24 h
	Urine ScACE	880.5*	83–777.8	mMol/24 h
	Urine AC/FC	1.94	0.42–3.04	/
	Plasma TC	58.5	36.2–72.9	nMol/mg protein
	Plasma FC	44.7	27.6–61.9	nMol/mg protein
	Plasma LcACE	9.2*	0.9–4.8	nMol/mg protein
	Plasma AC/FC	0.31	0.09–0.78	/
38 years	Urine TC	1538.0*	36–561.7	mMol/24 h
	Plasma TC	41.57	36.2–72.9	nMol/mg protein

*TC*, total carnitine, *FC* free carnitine, *AC* acyl carnitine, *ScCE* short-chain Acyl-carnitine ester, *LcCE* long-chain Acyl-carnitine ester. (*outside normal values)

### Genetic investigation

**Fig. 3:**
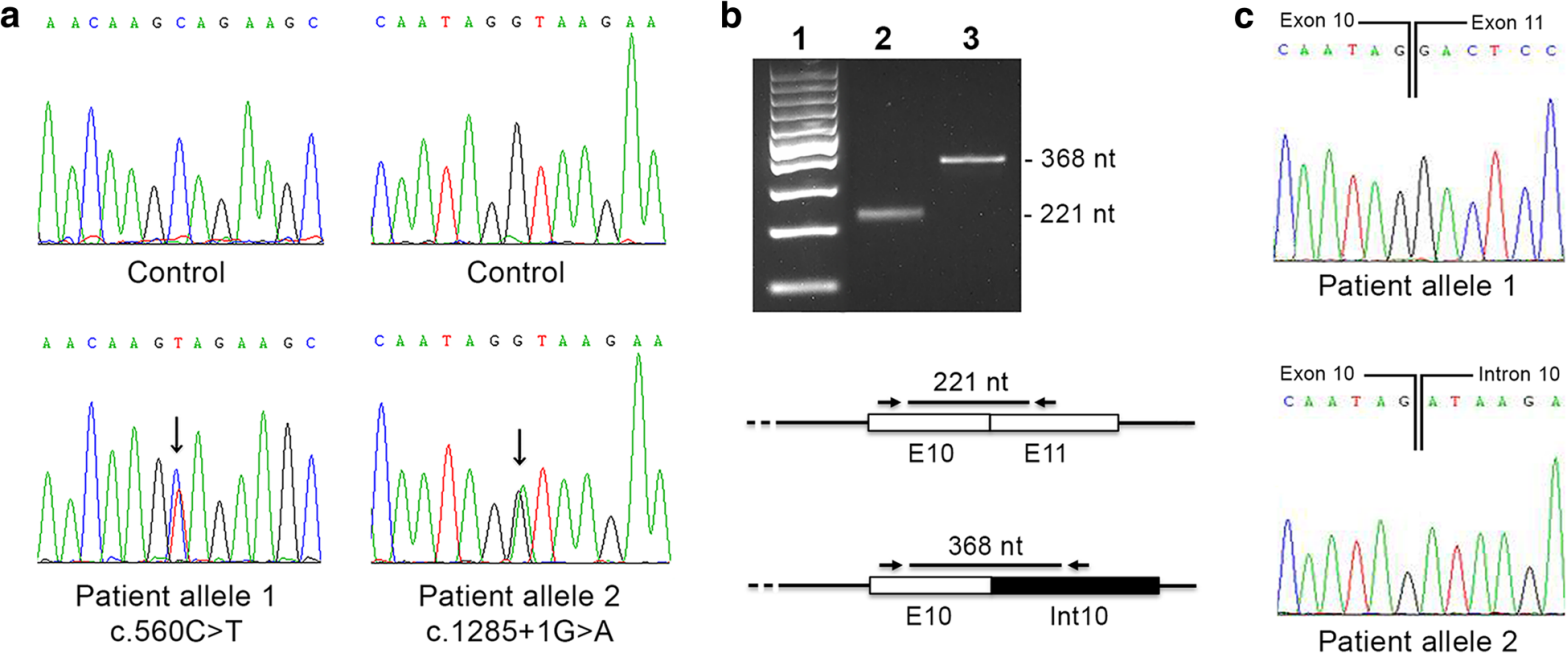
Molecular analysis of RR-MADD patient. **a** Electropherograms of *ETFDH* exon 5 harboring the c.560C > T mutation (allele 1) and exon 10 with c.1285 + 1G > A (allele 2), compared with control sequences; **b** RT-PCR from cDNA of RR-MADD patient. Two amplifications are performed using different primer pairs. The first reaction, with primers encompassing exons 10 and 11, detected wild type product (221 nt) in the allele 1; the second, carried out with oligonucleotides encompassing exon 10 and intron 10, shown a product of 368 nt, resulting from retention of intron 10. Lane 1: 100-bp molecular weight marker; Lane 2: patient allele 1; Lane 3: patient allele 2; **c** Electropherograms of 221 nt normal product (allele 1) and 368 nt aberrant product (allele 2)

Molecular analysis of the 13 coding exons and of the exon-intron boundaries of *ETFDH* gene revealed two mutations, in heterozygous status: the previously reported missense mutation, c.560C > T, [8], and a novel splice site mutation, c.1285 + 1G > A (GenBank accession number MH350092; Fig 3a). The first mutation, in allele 1, of *ETFDH* gene affects an aa residue localized within the second domain of the FAD region, p.A187V (Fig 1); the second variation, in allele 2, affects the invariant G of the intron-10 donor splice-site GT dinucleotide. This mutation is expected to lead to aberrant splicing with retention of intron 10. To verify whether c.1285 + 1G > A mutation disrupts RNA splicing, comparative *ETFDH* RT-PCR was performed from the peripheral blood of the patient using a forward primer localized in exon 10 and a reverse primer localized in intron 10. A 368 bp RT-PCR product was obtained from patient cDNA of allele 2, showing that intron 10 was not removed during RNA processing (Fig. 3b). Direct sequencing of the 368 bp RT-PCR product confirmed that patient cDNA contained the intron 10 sequence (Fig 3c). The c.1285 + 1G > A mutation resulted in a truncation of the *ETFDH* ORF at a premature stop codon located at the beginning of intron 10. The mutated protein is predicted to consist of 449 amino acids (G429Dfs21*).

### Bioinformatic studies of protein structure

**Fig. 4:**
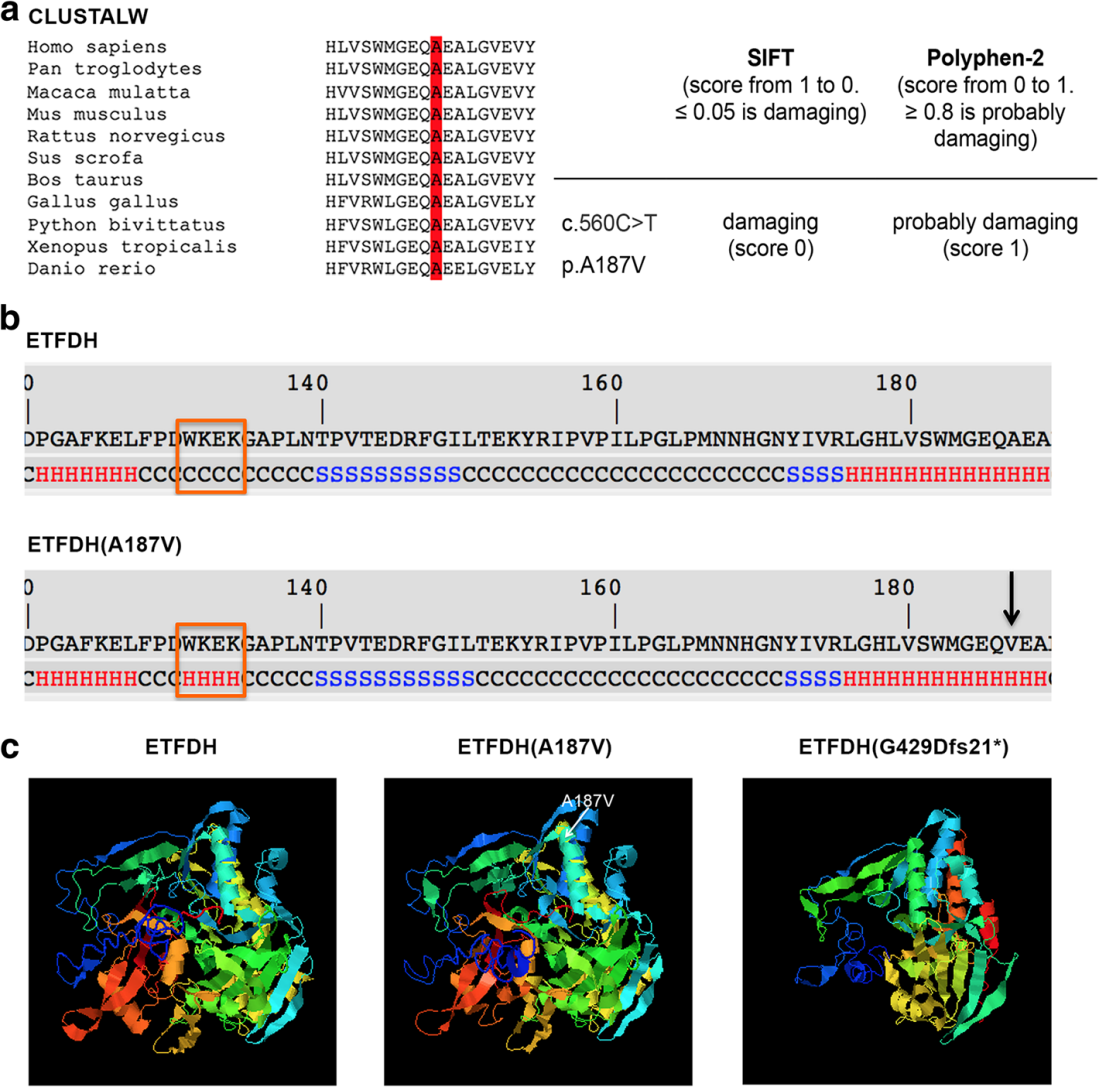
Structure characterization of ETFDH native and mutant proteins. **a** Sequence alignment among 11 vertebrates (*H. sapiens, P. troglodytes, M. mulatta, M. musculus, R. norvegicus, S. scrofa, B. taurus, G. gallus, P. bivittatus, X. Tropicalis, D. rerio*) around the site of A187V mutation. The A187 is located in a high conserved region; **b** Secondary structure of ETFDH(A187V) compared to ETFDH. Missense mutation determines a modification of structure from coiled coil (normal) to α-helix in RR-MADD patient at position 131–134 (indicated by rectangle). The A187V substitution is marked by arrow; **c** Predicted 3D model of ETFDH, ETFDH (A187V) and ETFDH (G429Dfs21*). The A187V (shown in central tridimensional model) generates conformational alterations of protein folding. The G429Dfs21* lacks the third segment of UQ-binding domain, second membrane-binding surface domain, second part of 4Fe4S cluster and C terminal domain, resulting in a misfolded protein

To clarify the pathogenetic charge of p.A187V, bioinformatic investigations were performed. Multiple alignments, produced by ClustalW comparing ETFDH protein sequences identified in eleven vertebrates, showed that A187 was localized in a highly conserved region (Fig. 4a); hence, it might affect ETFDH function. The analysis carried out using two predicted tools, PolyPhen-2 and SIFT, supported this hypothesis because both programs predicted a deleterious impact of p.A187V mutation on ETFDH protein (Fig. 4a).

Finally, to explore the impact of p.A187V and c.1285 + 1G > A mutations on protein function, the porcine ETFDH was used as model template in I-TASSER and SWISS_MODEL. Model obtained using I-Tasser shows that secondary structure of p.A187V displays a modification from coiled coil to α-helix between amino acids W131 and K134 (Fig. 4b). This conformational change can cause an incorrect folding of the mutant protein and can impair the ability of FAD binding (Fig. 4c). Similarly, the tridimensional structure prediction analysis of p.G429Dfs21* truncated protein revealed dramatic changes of ETFDH folding in comparison with the native conformation of the protein (Fig 4c).

## Discussion

MADD are multisystem genetic diseases characterized by various clinical manifestations with different degrees of severity. The most common clinical phenotype is the type III (RR-MADD), often associated with *ETFDH* gene mutations. In this study, we aimed to expand the spectrum of *ETFDH* mutations associated with RR-MADD and we obtained evidence of pathogenic effect of mutations reported here. We described a compound heterozygote subject presenting two *ETFDH* gene variations: a missense mutation, previously reported by us, [8] and a novel splice site mutation. The first mutation affects the amino acid residue 187 causing alanine to valine substitution. The amino acid A187 is located in the helix α3 of FAD motif. Mutations of residues localized in the helix α3 can have a significant impact on FAD binding [19]. Furthermore, analysis performed using I-Tasser software showed that A187V change modifies the secondary structure of four residues (W131-K134) located in the region that connects helix α2 and α3 of FAD region (Fig. 3b). As previously demonstrated, modifications involving amino acids of this segment can cause conformational alterations of FAD domain, decreasing protein stability and disrupting FAD binding activation of ETFDH [19]. The second mutation occurs in the donor site of intron 10 and affects RNA processivity by altering the splice site. Resulting protein lacks the C-terminal region comprising third segment of UQ-binding domain, the second membrane-binding surface domain and the second part of 4Fe4S cluster. It seems probable that the lack of part of UQ-binding region and of 4Fe4S cluster severely affects ETFDH-mediated electron-transfer pathway. Moreover, the absence of second membrane-binding surface domain could impair the correct localization of the protein into the mitochondrial membrane. Finally, bioinformatic prediction tools indicate that the loss of about 200 amino acids of native sequence give rise to a misfolded truncated protein.

The deleterious impact of both mutations, outlined by bioinformatics study, seems to correlate with the severity of clinical features of patient, who presented intermittent episodes of vomiting, acidosis and progressive muscle weakness since the infancy. At the age of 10 years she started a diet supplemented with MCT and carnitine. This treatment initially led to a good recovery of her condition. At the age of 36, a 200 mg of riboflavin was added, causing full and permanent improvement. In particular, the supplementation of riboflavin, a precursor of FAD, could have stabilized the structure of ETFDH(A187V) protein, partially restoring its activity. Some authors recommend also CoQ10 supplementation [20], but in this case it was not administered because our patient was already in good conditions.

Until now, about 700 MADD patients have been reported all over the world. 640 (95%) were affected by MADD [7, 9, 21–23]. It is well documented that many missense mutations in *ETFDH* impair FAD binding [10, 19]. FAD plays a central role in promotion of conformational stabilization and correct folding of many flavoproteins [24]. An increase of FAD concentration may restore stability of most of the ETFDH proteins carrying missense mutations. Some in vitro studies have been performed using fibroblasts obtained from MADD patients to test the stability and activity of ETFDH [10, 25]. In particular, fibroblasts of patients carrying different missense mutations of *ETFDH* (p.P456L, p.P483L and p.G429R), cultured with high concentrations of riboflavin in the medium, showed increase protein stability [10]. These variants lead to a milder impairment of native folding of ETFDH and the treatment with riboflavin partially restores enzymatic activity. Our patient is a compound heterozygote with a missense mutation and a truncated variation of *ETFDH*. The last mutation totally abrogates protein function, while we hypothesize that the p.A187V missense mutation induces structural defect, which partially destabilizes ETFDH folding. Riboflavin supplementation could have increased the stability and activity of ETFDH, resulting in the recovery of clinical symptoms. The same benefits have been reported in an other patient, put on restricted diet with riboflavin supplementation, who carried the A187V and the W343R mutations [8]. At the age of 38 she presented muscle weakness. The biopsy revealed lipid storage myopathy and the patient initially started a low-fat, high-protein diet with MCT and carnitine supplementation. Subsequently, after onset of muscle symptomatology, she added riboflavin treatment that produced marked improvement of muscle weakness. Regarding this patient, the later onset of MADD symptoms might be due to the presence of two missense mutations that maintain higher levels of ETFDH activity in comparison to our patient.

## Conclusion

In conclusion, we report here a new case of RR-MADD associated with mutations in *ETFDH* gene. Molecular analysis shows a previously described missense mutation and a novel splice alteration. Bioinformatic evaluation of ETFDH mutant proteins indicates that the missense mutation may induce conformational instability and a decrease of enzymatic activity, while the splice site mutation correlates with profound modifications of native folding, totally impairing protein function.

Finally, the data presented here support previous studies, showing that the treatment with riboflavin could prevent the dramatic catabolic failure that occurs in some subjects. Longitudinal studies are warranted to assess the efficacy of long-term riboflavin supplementation in RR-MADD patients.

## Abbreviations

CPK: Creatine phosphokinase
ETFDH: Electron transfer flavoprotein dehydrogenase
ETFα: Electron transfer flavoprotein α-subunit
ETFβ: Electron transfer flavoprotein β-subunit
FLAD1: Flavin adenine dinucleotide synthetase 1
LDH: Lactate dehydrogenase
LSM: Lipid storage myopathy
MADD: Multiple acyl-CoA dehydrogenase deficiency
MCT: Medium chain triglycerides
OX-PHOS: Mitochondrial respiratory chain enzymes activity
RR-MADD: Riboflavin-responsive multiple Acyl-CoA dehydrogenase disorder
SLC52A1: Solute carrier family 52 member 1
UQ: ubiquinone
